# Border control: selectivity of chloroplast protein import and regulation at the TOC-complex

**DOI:** 10.3389/fpls.2014.00483

**Published:** 2014-09-17

**Authors:** Emilie Demarsy, Ashok M. Lakshmanan, Felix Kessler

**Affiliations:** Laboratory of Plant Physiology, University of NeuchâtelNeuchâtel, Switzerland

**Keywords:** plastids, protein import, TOC complex, preproteins, post-translational modifications

## Abstract

Plants have evolved complex and sophisticated molecular mechanisms to regulate their development and adapt to their surrounding environment. Particularly the development of their specific organelles, chloroplasts and other plastid-types, is finely tuned in accordance with the metabolic needs of the cell. The normal development and functioning of plastids require import of particular subsets of nuclear encoded proteins. Most preproteins contain a cleavable sequence at their N terminal (transit peptide) serving as a signal for targeting to the organelle and recognition by the translocation machinery TOC–TIC (translocon of outer membrane complex–translocon of inner membrane complex) spanning the dual membrane envelope. The plastid proteome needs constant remodeling in response to developmental and environmental factors. Therefore selective regulation of preprotein import plays a crucial role in plant development. In this review we describe the diversity of transit peptides and TOC receptor complexes, and summarize the current knowledge and potential directions for future research concerning regulation of the different Toc isoforms.

## INTRODUCTION

Eukaryotic cells are composed of multiple compartments that acquire specialized sets of proteins for function. The vast majority of proteins are encoded by the nuclear genome. After synthesis in the cytosol accurate protein sorting and export toward their destination organelles rely on intrinsic topogenic sequences (Blobel, 1980). Initially, correct recognition of a preprotein requires specific receptors at the surface of the organelle. This crucial step of intracellular trafficking control can be viewed as a key–lock type mechanism.

Plant chloroplasts import impressive quantities as well as an enormous diversity of proteins from the cytosol. Large scale proteome studies indicate that 2000–4000 different proteins follow the chloroplast route (Ferro et al., 2003; Leister, 2003; Friso et al., 2004; Kleffmann et al., 2004). In the cytosol, chloroplast proteins are generally synthesized as preproteins with a N-terminal targeting sequence that is cleaved to produce the mature chloroplast protein upon import. This N-terminal targeting sequence, named transit peptide in the context of chloroplast protein import, faithfully guides the preprotein to the chloroplast surface where it engages the import machinery. In the following, the preprotein is translocated across the dual envelope membranes into the stroma. The transit sequence is cleaved upon arrival in the stroma yielding the mature form of the protein followed by folding in the stroma, targeting to the inner membrane via the conservative sorting pathway, or transport to the thylakoid membrane system. The recognition and translocation of the preprotein at the plastid envelope is provided by the TOC–TIC (translocon of outer membrane complex–translocon of inner membrane complex (TOC–TIC) import machinery. In pea, the core TOC complex consists of an assembly of the two GTP dependent receptors Toc34 and Toc159 together with the β-barrel protein conducting channel Toc75 (Hirsch et al., 1994; Kessler et al., 1994; Perry and Keegstra, 1994; Schnell et al., 1994; Becker et al., 2004). Upon engagement of the preprotein, the TOC complex associates with the TIC complex to form a continuous channel through the plastid envelope. The protein conducting channel at the TIC complex has been suggested to be made up of Tic110 or Tic20, or yet a combination of the two. Recently, however, it has been suggested that four core components form a 1MDa TIC channel [Tic20, Tic214 formerly known as YCF1, Tic56, and Tic100; (Kikuchi et al., 2013)]. Protein synthesis and targeting involve a large variety of cellular activities that are energy-requiring. Solely translocation of a single preprotein across the chloroplast envelope through the TOC–TIC machinery requires the hydrolysis of 650 ATP molecules on average, representing about 0.6% of the total light-saturated energy output of the organelles (Shi and Theg, 2013). Therefore a tight control of TOC–TIC mediated import activity is required to respect the cellular energy budget allocated to protein import.

Plants originate from a primary endosymbiotic event involving a photosynthetic cyanobacterium captured by a eukaryotic cell. The evolution of plants toward complex and multicellular organisms has been accompanied by the diversification of interconvertible plastid types displaying distinct and highly specialized biochemical and physiological functions (Jarvis and Lopez-Juez, 2013). For instance the most prominent plastid type, the chloroplast, develop from proplastid, or partially differentiated, non-photosynthetic etioplast, and can also differentiate into other non-photosynthetic plastid types such as chromoplast or elaioplast. Each plastid type requires the import of different subsets of proteins (Kleffmann et al., 2007; Brautigam and Weber, 2009; Barsan et al., 2012). Several strategies have evolved coordinately to ensure the selective import of plastid proteins. Together with the defined regulation of preprotein availability at the transcriptional levels, evolution also triggered diversification and increased complexity of both preprotein transit sequences (von Heijne and Nishikawa, 1991; Li and Teng, 2013) and composition of the import machinery (Reumann et al., 2005; Kalanon and McFadden, 2008; Gross and Bhattacharya, 2009; Shi and Theg, 2013). Evidence for the existence of different isoforms of TOC complex components has now been reported for several higher plant species including *Arabidopsis*, pea, and tomato (Jackson-Constan and Keegstra, 2001; Chang et al., 2014; Yan et al., 2014). Each isoform is thought to preferentially import a specific subset of client preproteins that may be the result of differential binding affinity (Jelic et al., 2003; Smith et al., 2004; Inoue et al., 2010; Dutta et al., 2014). Therefore, the relative abundance of Toc isoforms may reflect the protein composition of a given plastid type and be a key marker of plastid identity (Ling et al., 2012).

On top of that, plants are sessile organisms and need to adapt to ever-changing environmental conditions. Dynamic regulation of TOC complex composition may occur at the posttranslational level and represent a key regulatory mechanism contributing to the change in protein composition. By consequence this allows rapid modulation of plastid metabolism to ensure and drive plant development and acclimation. Thus, the relative abundance of Toc receptor may not only be a marker of plastid type but also of plastid state (Agne et al., 2010; Ling et al., 2012).

The molecular mechanisms underlying the process of protein translocation have been reviewed extensively (Jarvis, 2008; Andres et al., 2010; Li and Chiu, 2010). Here, we present the current knowledge with regard to the selectivity and the regulation of the preprotein import process at the level of the TOC complex.

## PREPROTEIN IMPORT IN PLASTID IS REGULATED BY DEVELOPMENTAL AND ENVIRONMENTAL FACTORS

Years before the identification of any of the components of the chloroplast protein import machinery (Dahlin and Cline, 1991) proposed that import activity is correlated with protein demands during plastid development. They observed a high import activity in non-photosynthetic proplastids, which gradually decreased as plastids matured. This phenomenon was observed for etioplast as well as chloroplast development. Interestingly, when dark-grown plants were shifted from dark to light the import activity of etioplasts was activated to accommodate the set of preproteins required for chloroplast differentiation (Dahlin and Cline, 1991). This seminal study focused on a few substrates and, given the experimental limitations at the time, was unable to provide a complete picture of plastid protein import dynamics. Recently, this topic was reinvestigated using a larger number of chloroplasts precursors proteins (Teng et al., 2012). This study confirmed that preprotein specificity is modulated in synchrony with chloroplast developmental stages. Interestingly, this study demonstrated that the earlier results by Dahlin and Cline (1991) cannot be extended to all import substrates. Rather, Teng et al. (2012) refined the model and classified the substrates according to their importability in chloroplasts at different developmental stages and consequently defined three age-selective classes: substrates that are imported more efficiently in young chloroplasts (group I), in older chloroplasts (group III), whereas group II represents substrates that are imported similarly in developing and mature chloroplasts. Thus, it appears that regulation of chloroplast preprotein import is part of a differential age-specific regulatory network.

*In vitro* import experiments using different isolated plastid types as well as the visualization of protein targeting using transgenic lines expressing transit peptides fused to GFP support the notion that import selectivity is regulated in a tissue specific manner (Wan et al., 1996; Primavesi et al., 2008; Yan et al., 2014). Finally temperature stress (cold and heat) on intact pea leaves and isolated chloroplasts was found to reduce import of the small subunit of RubisCO preprotein (pSSU; Dutta et al., 2009).

In summary, these results demonstrate that both plastid import activity and selectivity are modulated in accordance with plastid type, developmental stage, and environmental condition. For this purpose plants have evolved a complex set of preprotein import components with specialized features and regulatory mechanisms (Jarvis et al., 1998; Kubis et al., 2004).

## PREPROTEIN SELECTIVITY AT THE CHLOROPLAST IMPORT MACHINERY

### OVERVIEW OF THE TOC–TIC MACHINERY

The TOC-TIC pathway (translocon of outer membrane complex-translocon of inner membrane complex) is the major protein import pathway in higher plants (Bauer et al., 2000; Asano et al., 2004; Kovacheva et al., 2005). Most of the proteins with cleavable transit peptides that are targeted to the stroma, thylakoid membranes and lumen follow this route, that is therefore vital for plastid biogenesis (Kessler and Schnell, 2006; Bischof et al., 2011; Dutta et al., 2014). The native TOC–TIC complex in pea and *Arabidopsis* has been found to include two GTPase-receptors Toc159 and Toc34, a channel protein Toc75 and at least three additional regulatory Toc proteins Toc64, Toc22, and Toc12 (Andres et al., 2010). At the inner membrane at least 11 different proteins have been reported to be involved in the import process (Kovacs-Bogdan et al., 2010; Kikuchi et al., 2013). Electrophysiological experiments suggested that Tic110 and Tic20 could function as channels facilitating the translocation of preproteins across the inner membrane (Kikuchi et al., 2009, 2013; Kovacs-Bogdan et al., 2011). These two channels are thought to function independently and in different complexes (Kikuchi et al., 2009, 2013; Kovacs-Bogdan et al., 2011). This is supported by the finding that Tic110 interacts with preproteins and TOC complexes (Schnell et al., 1994; Inaba et al., 2005) but not with Tic20 (Kikuchi et al., 2009). Tic110 is a protein of eukaryotic origin present in various plastid-containing organisms (Shi and Theg, 2013). Its function is indispensable for plant viability and chloroplast biogenesis (Inaba et al., 2005). Based on these data it was proposed that Tic110 has an essential role in chloroplast protein import. Recently, composition of the Tic20 complex in *Arabidopsis* has been investigated using Blue Native PAGE and mass spectrometric analyses. The results suggested that Tic20 associates with Tic56, Tic100, and Tic214 (Kikuchi et al., 2013). Although Tic20 is of prokaryotic origin and is well conserved among the plant kingdom, Tic56, Tic100, and Tic214 appear to have specifically evolved in a limited number of higher plant species only (Kikuchi et al., 2013). Tic214, also known as YCF1, is absent from the genome of some Poacae species (Jensen and Leister, 2014; Smith and Lee, 2014), thus the role of TIC20 complex as the general inner chloroplast membrane translocon in higher plants is questionable. Nevertheless the albino, seedling lethal phenotype of null mutants of each of the TIC20 complex subunits underscores their functional importance at least in *Arabidopsis*. In conclusion the exact contribution of TIC110 and TIC20 complexes in chloroplast protein import is still under debate.

At the evolutionary level, a view of growing complexity of the composition of TOC machinery is emerging (Shi and Theg, 2013). Starting with one channel protein at the outer envelope in cyanobacteria, the outer envelope protein import complex has evolved into a GTP-regulated multi-protein complex in higher plants (Olsen and Keegstra, 1992; Schnell et al., 1994; Hiltbrunner et al., 2001a; Kessler and Schnell, 2002; Voulhoux et al., 2003). The Toc receptors can form homo- and heterodimers in a dynamic way regulated by preprotein binding and GTP binding/hydrolysis activity (Smith et al., 2002; Sun et al., 2002; Wallas et al., 2003; Lee et al., 2009b; Rahim et al., 2009; Oreb et al., 2011). Although GTP binding and GTPase activity seem dispensable (expression of GTPase/dimerization-defective Toc159 and Toc33 complement the corresponding knock out mutants), it has been shown that they are required for full preprotein import efficiency *in vitro* (Agne et al., 2009; Aronsson et al., 2010; Aronsson and Jarvis, 2011). In most higher plants the Toc75 channel is encoded by a single gene (Inoue and Keegstra, 2003), but normally more than one homolog for the plastid specific GTPase families Toc159 and Toc34 exists, and thus there is the possibility of making various combinations of TOC complexes (Hiltbrunner et al., 2001a; Chang et al., 2014; Yan et al., 2014). The evolution of a translocation route depending on GTP-binding as well as other accessory proteins may be seen as the key to the developmental stage specific regulation of protein import in higher plants (Schleiff and Soll, 2005; Gagat et al., 2013).

### DIVERSITY AND FUNCTIONAL SPECIFICITIES OF TOC GTPase RECEPTORS

**FIGURE 1:**
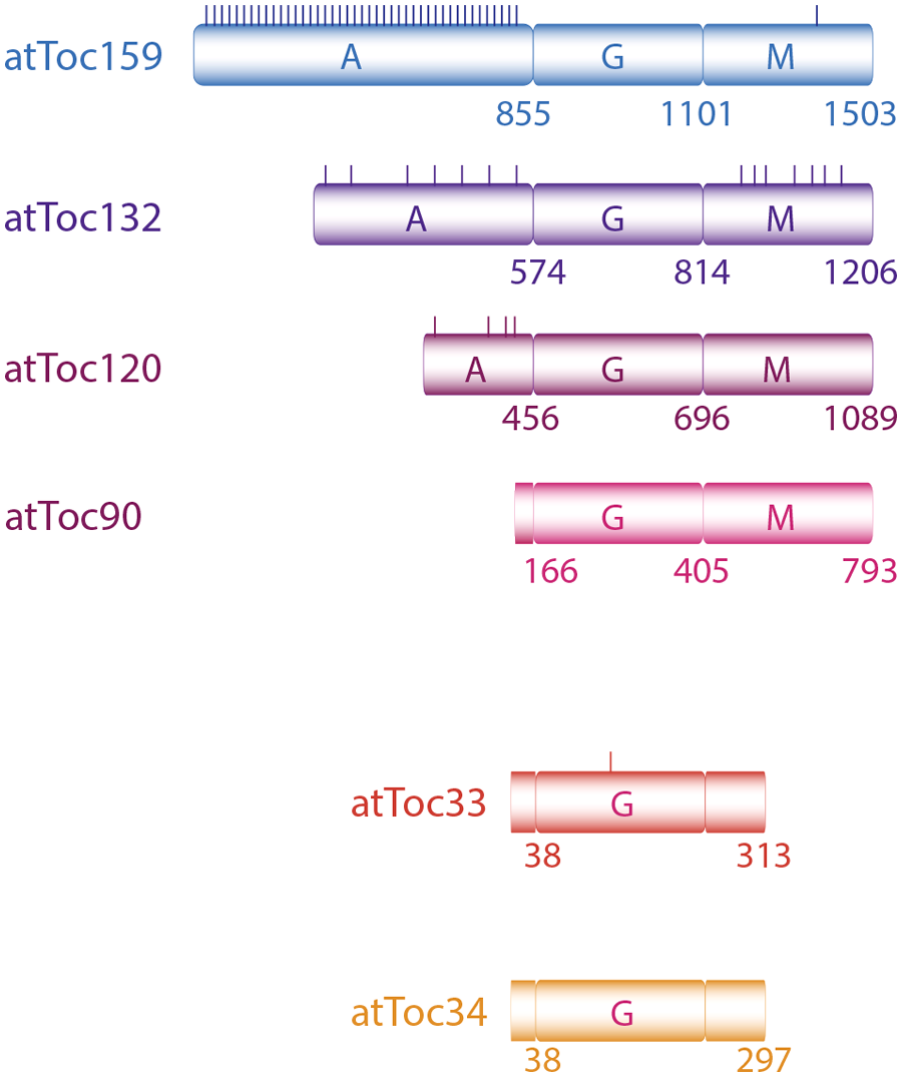
**The translocon of outer membrane complex includes two GTPase-receptors Toc159 and Toc34.** Toc159 consists of a GTPase domain (G) flanked by a C-terminal membrane anchoring domain (M) and an acidic N-terminal region (A). In *Arabidopsis* four Toc159 and two Toc34 isoforms have been identified. Toc159 homologs differ primarily in their A-domain sequences and lengths. Experimentally identified phospho-serine and -threonine residues (PhosphAT 4.0) are schematically indicated by short vertical lines.

Members of Toc159 family are characterized by three distinct domains: M- (membrane anchoring) domain, G- (GTP-binding) domain, and a highly acidic, intrinsically disordered A-domain (**Figure 1**). There are four homologs in *Arabidopsis thaliana*: atToc159, -132, -120, and -90. While they share high similarity in their G- and M-domains, they largely differ in length and sequence at their A-domains (Jackson-Constan and Keegstra, 2001; Hiltbrunner et al., 2001a). Toc34 proteins are smaller, membrane-anchored GTPases. In pea, only one member has been detected so far while two isoforms of Toc34 (atToc34 and atToc33) have been identified in *Arabidopsis* (Jarvis et al., 1998; Gutensohn et al., 2000).

Genetics and biochemical studies have supported the idea that various combinations of the different Toc GTPase isoforms lead to a diversity of complexes displaying differential selectivity for preprotein recognition and translocation (Kubis et al., 2003, 2004; Constan et al., 2004; Ivanova et al., 2004). Co-immunoprecipitation experiments performed by Ivanova and collaborators demonstrated that atToc159 preferentially associates with atToc33, while atToc120, and/or atToc132 preferentially form a complex together with atToc34 (Ivanova et al., 2004). Interestingly, the *toc34 (ppi3)* knock out mutant has no visible defect, while the *toc33 (ppi1)* mutant displays a pale green phenotype with a chloroplast biogenesis defect similar (although much less severe) than the *toc159* mutant phenotype (*ppi2*), supporting the proposition that these latter two receptor isoforms function in the same complex and preprotein import pathway (Jarvis et al., 1998; Bauer et al., 2000; Kubis et al., 2003, 2004; Constan et al., 2004).

Several lines of evidence indicate a potential functional overlap of the two Toc34 members: the strong sequence similarity: 65% (Jarvis et al., 1998); the fact that a minor fraction of atToc33 was co-immunoprecipated with Toc120/132, and atToc34 was detected with atToc159 (Ivanova et al., 2004); the embryo lethal phenotype of *toc33/toc34* double mutants and, most importantly, the ability of atToc34 to complement *ppi1* phenotype (Jarvis et al., 1998; Kubis et al., 2003; Constan et al., 2004). Transgenic complementation studies also indicated the potential functional overlap of atToc120 and atToc132 (Ivanova et al., 2004; Kubis et al., 2004) and, to a limited extent, for atToc159 and atToc90 (Infanger et al., 2011), however, no functional overlap exists between these two subgroups [atToc120/132 vs. atToc159/atToc90 (Ivanova et al., 2004; Kubis et al., 2004)]. While the two Toc34 homologs are mutually exchangeable, the same is only partially true for the Toc159 homologs, suggesting that preprotein selectivity of TOC complexes is mostly conferred by the identity of the Toc159 isoforms.

The classification of the client proteins of each isoform has been attempted. Because of the albino phenotype of *ppi2*, it has been proposed that Toc159 primarily facilitates the import of photosynthesis-associated preproteins. On the other hand, Toc132, or Toc120 being present predominantly in roots could facilitate that of constitutive (housekeeping) preproteins (Kubis et al., 2003, 2004; Ivanova et al., 2004; Smith et al., 2004; Inoue et al., 2010). *In vitro* import assays using a selection of substrates support this model (Smith et al., 2004; Inoue et al., 2010). However, the albino phenotype of the *ppi2* mutant was shown to result not only from a defect in the import of a set of chloroplast proteins, but also from the transcriptional downregulation of a specific set of nuclear genes associated with photosynthesis (Bauer et al., 2000; Kakizaki et al., 2009). This effect is commonly referred to as retrograde signaling, and pleiotropically affects albino and pale green mutants across the board. The interference of retrograde signaling with preprotein import in *ppi* mutants has blurred the identification of the specific substrates of each of the receptor isoforms. Comparative analysis of *ppi2* mutant proteome and transcriptome demonstrated that certain photosynthesis-associated proteins accumulated normally in plastids even in the absence of atToc159, whereas accumulation of some house-keeping proteins were strongly diminished despite their mRNA expression levels being similar to the wild type (Bischof et al., 2011). Furthermore, the results of a yeast two hybrid screen used to identify the preferred Toc receptor of a variety of preproteins supported to the finding of (Bischof et al., 2011; Dutta et al., 2014). Together these studies affirmed that Toc GTPases, especially the Toc159 homologs, confer specificity to plastid preprotein import. However, specificity is not likely to be based on the photosynthetic or housekeeping nature of a preprotein. This is a move away from the overly simplistic paradigm of “photosynthesis-associated” and “house-keeping” specificities toward a more differentiated model that reflects complex and varying plastid preprotein requirements during development and under environmental influence. Therefore, Toc client protein classification will need to be rethought along these lines. One hypothesis is that the combination of preprotein specificities of plastid resident Toc receptors reflects the tissue or cell specific preprotein accumulation patterns that are specific to a particular plastid type.

As mentioned above Toc159 homologs diverge the most at their A-domains, suggesting a key role in their functional specialization. In domain swapping experiments, Inoue et al. (2010) replaced the A-domain of atToc132 by that of atToc159. Expression of this construct partially restored chlorophyll accumulation in the *toc159* null mutant (*ppi2*), while no complementation was observed using a construct encoding atToc132 without an A-domain. These data elegantly demonstrated that the functional specialization relies at least partially on intrinsic properties of the A-domain (Inoue et al., 2010). In agreement with this, it was observed that removal of the A-domains of atToc159 and atToc132 reduced the binding selectivity of these isoforms (Smith et al., 2004; Inoue et al., 2010; Dutta et al., 2014). Apparently, the A-domain does not directly interact with preproteins but may act as a filter enhancing the affinity for subsets of proteins and reducing the affinity for others (Dutta et al., 2014). Preprotein binding to Toc159 has been shown earlier to occur at the G-domain (Smith et al., 2004). Thus it seems likely that the A-domain influences the G-domain by, for instance, positively, or negatively modulating access of a preprotein according to its nature. Finally, the lack of complementation of *ppi2* by atToc132 lacking an A-domain (Inoue et al., 2010) as well as the recent work of Smith et al. (2004) using a yeast two hybrid system to study the preprotein-Toc159 receptor isoforms affinity (Dutta et al., 2014) indicate that a degree of specificity is conferred by the G-domain itself.

### DIVERSITY AND COMPLEXITY OF THE TRANSIT PEPTIDES

Inherently, recognizable specificity features would need to be encoded in the plastid transit peptides. One general consideration regarding the transit peptides is that no consensus can be defined, even when considering the structure at the three dimensional level (von Heijne and Nishikawa, 1991; Bruce, 2001). Plastid transit peptides largely vary in length from an average of 50 up to 146 amino acids (Li and Teng, 2013). There are some features shared with mitochondrial targeting peptides such as the overrepresentation of serine and threonine residues that may explain the targeting of plastid transit peptide containing proteins to mitochondria when expressed in heterologous animal systems (Zhang and Glaser, 2002). No further similarities between plastid and mitochondrial targeting sequences have been identified, and other levels of specificity might exist and enable plant cells to discriminate and accurately sort the two types of organellar proteins. Interestingly, an estimated thirty percent of chloroplast localized proteins do not have a canonical transit peptide (Ferro et al., 2003; Leister, 2003; Kleffmann et al., 2004, 2007; Jarvis, 2008). A recent study in pea indicated that this may be an overestimation that results from a slightly inaccurate algorithm that does not take into account the whole diversity of features of plastid transit peptides (Chang et al., 2014).

The diversity of transit peptides sequences might well be explained by the need to fine tune the import of specific subsets of proteins in agreement with plastid type and developmental stage. Toc159 binds preproteins via their N-terminal, transit peptides (Smith et al., 2004), so one might reasonably expect that the specificity determinants reside within this particular region. However, the determining sequence elements that confer selectivity to a Toc159 isoform have not yet been identified. They could consist of cryptic signals buried in motifs and multiple-motifs (Lee et al., 2009a; Bionda et al., 2010; Chotewutmontri et al., 2012). For example Lee et al. (2009a) revealed that Toc159-dependent import can be mediated by multiple independent motifs, one that consists in a stretch of serine residues located in first 12 amino acid of the N-terminal region of preRBCS (pSSU), and one located in the C-terminal part of the transit peptide sequence (Lee et al., 2009a). In a recent review, (Li and Teng, 2013) analyzed such motifs and their relation with binding sites for various proteins involved in preprotein import. The authors then attributed the preproteins to distinct subgroups based on patterns of sequence motifs in combination with their capacity to be targeted and bind to the protein translocon at the chloroplast outer envelope. Though only a limited number of preproteins were taken into account in these analyses, they clearly indicated that complexity of transit peptide design plays a key role in import selectivity.

## REGULATION OF TOC COMPONENTS

### EXPRESSION PATTERN

Regulation of TOC complex activity occurs at several levels. Overall the accumulation levels of Toc components throughout development appear to reflect the total import activity, i.e., a highest level of expression for the different components is observed in young, developing tissue, as compared to mature organs (Jarvis et al., 1998; Yu and Li, 2001; Kubis et al., 2003, 2004; Ivanova et al., 2004). As an exception, Toc90 appeared to be uniformly expressed throughout development (Kubis et al., 2003; Infanger et al., 2011). Specific patterns were revealed when comparing the expression levels of the different Toc receptors isoforms in different organs and/or different plastid types, and usually correlated with corresponding mutant phenotypes in *Arabidopsis* (Jarvis et al., 1998; Bauer et al., 2000; Gutensohn et al., 2000; Kubis et al., 2004; Yan et al., 2014). atToc159 and atToc33 are the most highly expressed members of their respective families and both mutants displayed the most severe visible phenotype when compared to other single mutants (Jarvis et al., 1998; Kubis et al., 2004). Furthermore, defects of plastid development in the corresponding mutants follow the expression pattern of the corresponding gene: highly regulated expression is observed for atToc159 and atToc33, with a higher expression occurring in photosynthetic tissues, when compared to other family members. Accordingly single mutants of these genes are specifically affected in plastid type present in those tissues, i.e., the chloroplast and its precursor, the etioplast (Jarvis et al., 1998; Bauer et al., 2000). By the same token, the higher expression of atToc120 and atToc132 in roots correlates with a severe defect of root plastid development in the corresponding double mutant (Kubis et al., 2004). Similarly the mutant phenotype of atToc34, which is expressed more highly in roots, retains normal plastid development but displays reduced root length (Gutensohn et al., 2000; Constan et al., 2004). Thus, selectivity of import into plastids can be modulated at least in part by transcriptional regulation of Toc components in accordance with plant tissue and/or growth conditions (light conditions in the case of Toc159).

**FIGURE 2:**
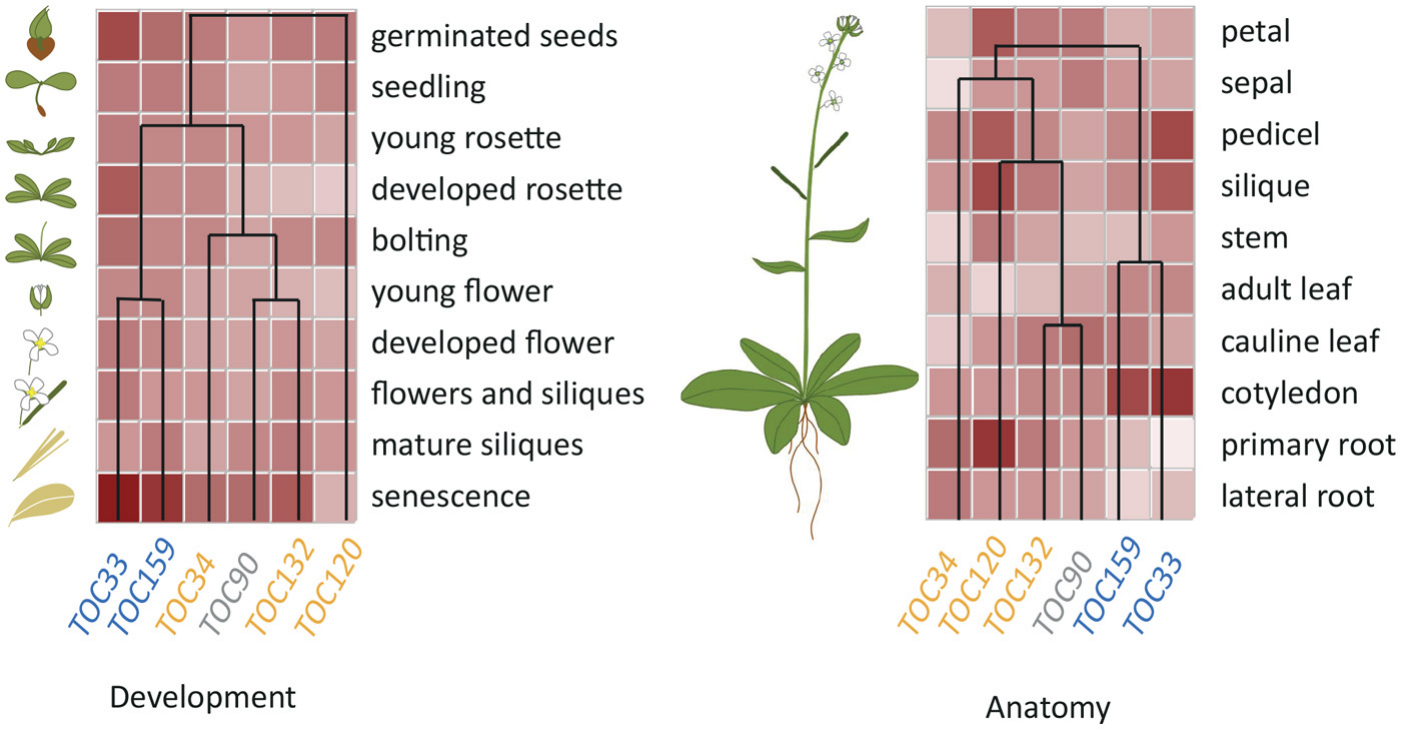
**Hierarchical Cluster analysis of *Arabidopsis* Toc receptor gene expression.** TOC complexes consist of the assemblies of two different receptors from two separate GTPase families, Toc159/-132/-120/-90 and Toc33/-34, respectively, together with the Toc75 channel. Biochemical and genetic evidence have shown that atToc159 preferentially associates with atToc33 whereas atToc132 and 120 preferentially associate with atToc34. These specific associations are reflected by co-regulation of the Toc receptors isoforms. Data were extracted from Genevestigator database (Nebion), using the Hierarchical Cluster analysis tool, with “Development” or “Anatomy” specific selections for left- and right-hand panels, respectively.

Expression profiles of the different Toc members suggest that the receptors acting together in a specific complex are co-regulated at the transcriptional levels. Interestingly, hierarchical cluster analysis indicates that this co-regulation extends to a large variety of conditions (**Figure 2**) and suggests that common *cis* and *trans* regulatory elements could regulate associated Toc receptors. In support of this idea, the CIA2 transcription factor was found to co-modulate atToc33 and atToc75 expression specifically in leaves (Sun et al., 2001, 2009). However, the identity of other transcription factors responsible for the differential expression of Toc members has been poorly investigated so far and further experimentation will be necessary to reveal the molecular mechanisms underlying the regulation of Toc gene expression.

### POST-TRANSLATIONAL MODIFICATIONS

**FIGURE 3:**
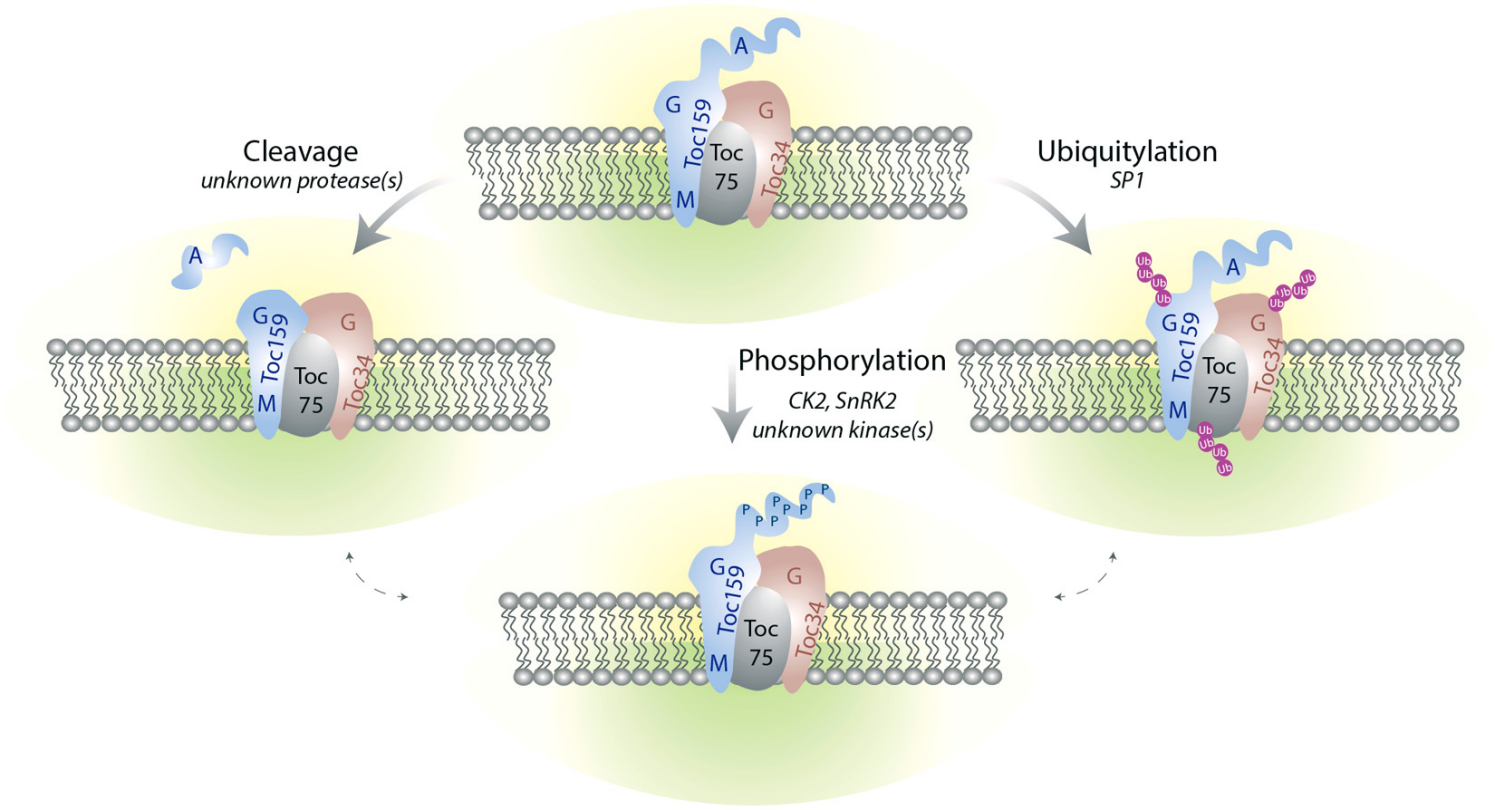
**The TOC complex is targeted by multiple post-translational modifications.** Phosphorylation of Toc159 and Toc34 at the G-domain may regulate homo and heterodimerization of the Toc receptors as well as their interaction with preproteins. The A-domain of Toc159 is hyperphosphorylated and can be released from the rest of the protein by proteolysis. The functional significance of these A-domain modifications are unclear but they may modulate the selectivity of the receptors for their client preproteins. All the Toc components are subject to ubiquitylation. Ubiquitylation may serve as a signal for proteasome-mediated degradation and pave the way for remodeling of TOC complex composition during plastid differentiation or environmental adaptation. The signaling pathways as well as the environmental and/or developmental factors triggering these PTM remain poorly described. Whether crosstalk between the different types of regulation exists is also not yet known.

Differential regulation of Toc components also occurs at the post-translational levels (**Figure 3**). It is interesting to note that the *ppi2* mutant can be complemented by expression of atToc159 under the constitutive 35S promoter indicating that transcriptional regulation can be bypassed at least under laboratory conditions (Kubis et al., 2004; Agne et al., 2009).

#### Phosphorylation

Several studies have shown that Toc receptors are phosphorylated. Phosphorylation has been reported for pea Toc34 and its ortholog atToc33 (Ser113 and S181, respectively), while it was not detected for atToc34 (Sveshnikova et al., 2000; Fulgosi and Soll, 2002; Jelic et al., 2002, 2003). Differential phosphorylation could therefore represent a regulatory mechanism conferring specificity to the two different members of *Arabidopsis* Toc34 family.

*In vitro* studies indicated that phosphorylation has a negative effect on GTP and preprotein binding to psToc34 and atToc33 (Sveshnikova et al., 2000; Jelic et al., 2003). Furthermore, *in vitro* and *in vivo* data showed that phosphorylation/phosphomimicking at atToc33 and phosphorylation of psToc34 negatively influenced TOC complex integrity (Oreb et al., 2008). Hypotheses for the underlying molecular mechanisms have been put forward. Since GTPase activity may be required for G-domain-mediated association of Toc159 and Toc34 (Smith et al., 2002; Wallas et al., 2003), phosphorylation may indirectly prevent homo- as well as heterodimerization because of a negative effect on GTP-binding. More directly the bulky, negatively charged phosphate group could inhibit the binding to a preprotein or to Toc159. However, this latter hypothesis may be valid for *Arabidopsis*, but not for pea since the phosphorylation site is distant from the dimerization interface (Oreb et al., 2008). In summary, the available data suggest the phosphorylation of psToc34 and atToc33 have a dual function, regulating both TOC complex assembly and subsequent substrate binding.

The physiological relevance and the signals triggering this specific phosphorylation are still not clearly defined. Data obtained from *Arabidopsis* transgenic lines expressing phosphomicking variants of atToc33 confirmed that phosphorylation at S181 can inhibit atToc33 activity in young *Arabidopsis* seedlings but not later during development (Aronsson et al., 2006; Oreb et al., 2007). Indeed, phosphomimick variants resemble the *ppi1* mutant regarding a number of phenotypic traits in 5 day-old *Arabidopsis* seedlings (chlorophyll accumulation, chloroplast ultrastructure, and photosynthetic activity). However, since the non-phosphorylatable version behaved similarly to the WT, it was not possible to determine the conditions under which atToc33 is phosphorylated in *planta* (Aronsson et al., 2006; Oreb et al., 2007). We speculate that phosphorylation might represent a means to quickly down-regulate preprotein import *via* atToc33 containing TOC complexes, for example in mature plastids where protein demand is low. Moreover and since atToc33 can be phosphorylated but not atToc34, this post-translational regulation may affect the selectivity aspect of preprotein import regulation.

One additional phosphorylation site has been experimentally identified in both atToc33 and -34 [data provided by PhosphAT (Durek et al., 2010)]. It maps to a conserved Tyrosine residue of the G-domain. Additional studies will be required to validate and determine the regulatory effect of this specific phosphorylation.

Finally, the identity of Toc33/Toc34 kinase(s) still remain(s) mysterious. Some clues stemming from pea suggest that psToc34 is phosphorylated by an ATP-dependent, 98 kDa kinase residing at the outer envelope membrane (Fulgosi and Soll, 2002). However, the amino acid sequence information is not sufficient to molecularly identify the potential kinase in pea or its homolog in *Arabidopsis*.

The Toc159 receptors are also targets of phosphorylation. First evidence of phosphorylation of Toc159 came from *in vitro* studies using outer envelopes isolated from pea chloroplasts, showing that both full length Toc159 and its natural 86 kDa fragment could be phosphorylated (Fulgosi and Soll, 2002). Phosphorylation was demonstrated for the G-domain of psToc159, reminiscent of Toc33/34 regulation (Oreb et al., 2008), however, neither the precise site nor the regulatory function were further investigated. Large-scale phosphoproteomics projects revealed that Toc159 members in *Arabidopsis* are highly phosphorylated at the acidic A-domain (Agne et al., 2010; Durek et al., 2010). In total, 43 sites have been mapped in atToc159, while far fewer were detected in the other three members. These lower numbers may be due to the shorter length of the atToc132 and atToc120 A-domains, the absence of such a domain in atToc90, or because lower protein accumulation levels when compared to atToc159 limit the detection by mass spectrometry. Nevertheless the identified phosphorylation sites do not map to matching positions in the different homologs, which confers an additional degree of divergence to the A-domain.

The functional relevance of A-domain phosphorylation has been poorly documented so far. The dispensable nature of the A-domain suggests that phosphorylation either plays a minor role altogether, or possibly an important regulatory role under specific conditions (Hiltbrunner et al., 2001b; Agne et al., 2009; Inoue et al., 2010). The A-domain behaves as an intrinsically disordered protein, which is often linked to multiple and transient protein–protein interactions (Richardson et al., 2009). Therefore phosphorylation of this domain could modulate interactions of Toc159 with other Toc components but also with specific sets of client preproteins. In addition, a selective autoinhibitory function of the A-domain under specific conditions may be envisaged that may be alleviated by phosphorylation or proteolytic removal.

Recently a link between ABA signaling and phosphorylation of Toc159 family members in *Arabidopsis* has been established (Wang et al., 2013). Upon ABA treatment atToc159 was phosphorylated at Thr692. atToc120 and atToc132 phosphopeptides accumulation was also enhanced by ABA treatment. These data together with the fact that a mutant deficient in ABA synthesis is affected in pre-protein import and early plant development suggest a close link between ABA signaling and chloroplast protein import regulation via Toc159 A-domain phosphorylation (Zhong et al., 2010). Whether ABA dependent phosphorylation plays a role in preprotein recognition, impacts TOC159 complex assembly, or acts at the level of the translocation process will be interesting questions to be addressed in the future.

Several classes of kinases may mediate phosphorylation of Toc159 homologs. Motif analysis suggests that a large fraction of atToc159 phosphorylation sites represent potential cytosolic casein kinase 2 (CK2) targets and this was validated biochemically by *in vitro* phosphorylation experiments (Agne et al., 2010). Recently it has been shown that ABA dependent phosphorylation of atToc159 at Thr692 was decreased in a triple mutant *snrk2.2/2.3/2.6* that is nearly insensitive to ABA treatment (Wang et al., 2013). In addition SnRK2.6 phosphorylated recombinant atToc159 *in vitro*. Thus SnRK2.6 represents a potential kinase of atToc159 at Thr692. On the contrary, atToc120 and atToc132 phosphorylation upon ABA treatment was detected only in the triple mutant *snrk2.2/2.3/2.6*, indicating the involvement of another ABA regulated kinase. Indeed ABA signaling is mediated by multiple kinases of the SnRK family but also of the MAPK kinase family (Danquah et al., 2014). The phosphorylation status of Toc159 members could therefore be regulated antagonistically by ABA signaling via the action of different classes of kinases and could represent a way to switch between Toc132/Toc120 and Toc159 specific import depending on environmental as well as developmental conditions and consequent plastid preprotein requirements. Finally, it has been proposed that psToc159 is a target of a 70 kDa kinase located at the outer envelope of the pea chloroplast (Fulgosi and Soll, 2002) but so far no study has reported on the identification of a putative homolog in *Arabidopsis*.

In conclusion phosphorylation of the Toc159 and Toc34 receptors potentially regulates protein import at different levels: it may impact the import rate by regulating the affinity toward client preproteins, or affect the composition of the TOC complex by modulating the interaction between Toc receptors and consequently change the selectivity of plastid protein import. The involvement of ABA signaling in this regulation indicates that phosphorylation of Toc components can modulate the import activity in response to developmental signals for example during germination or subsequent post-germinative processes, or in response to abiotic stress that require the tuning of the plastid proteome. Hormonal control of plastid development has been frequently reported, but the effects on import activity are still poorly documented.

Phosphorylation could also be part of a signaling cascade enabling subsequent additional post-translational modifications (PTM) since cross talk between different is a common phenomenon in eukaryotic systems, and PTM other than phosphorylation have been described for the different Toc components (see below). The existence of numerous phosphorylation sites, especially in Toc159 families, suggests the participation of multiple kinases, and corresponding signaling pathways probably acting in a network.

#### Post-translational modifications other than phosphorylation

Toc159 was first identified as an 86 kDa protein lacking the A-domain (Hirsch et al., 1994; Kessler et al., 1994; Schnell et al., 1994; Bolter et al., 1998). It is not clear whether proteolysis occurs only during chloroplast preparation or whether it is part of regulatory system acting on Toc159. It is not clear either if other Toc159 homologs are also substrates of proteolytic cleavage but the relative stability of the A-domain fragment of atToc159 favors controlled proteolysis (Agne et al., 2010). Therefore, a yet unknown protease may process Toc159 conditionally, leading to the removal of the A-domain and consequently altering the import selectivity. Interplay between phosphorylation and cleavage has been demonstrated in other biological systems for example in the context of apoptosis (Dix et al., 2012). Investigation of the cross talk between these two PTM will certainly be an interesting aspect for future research.

Abundance of the different Toc members varies developmentally. Currently an important question is to understand how the TOC machinery is remodeled upon plastid development and plastid inter-conversion. As discussed above transcriptional regulation plays a role in modulation of Toc components expression depending on plant tissues and environmental conditions, while PTM may participate in the regulation of TOC complex assembly and activity. Recently a genetic study complemented by biochemical analyses revealed that Toc receptors as well as the Toc75 channel could be modified by ubiquitylation. Ubiquitylation required SP1, a chloroplast outer membrane localized E3 ubiquitin ligase (Ling et al., 2012). Enhanced accumulation of TOC proteins in *sp1* genetic background suggested that SP1 indeed participates in UPS-mediated degradation of Toc components. Phenotypic analyses indicated that this regulatory mechanism may play a role during plastid inter-conversion. However, how SP1 is regulated and functions selectively on the different Toc receptors has not been addressed so far. Again a possible interplay with phosphorylation regulation might be envisaged as phosphorylation can serve as either a positive or a negative signal for ubiquitylation (Hunter, 2007).

## CONCLUDING REMARKS

Acquisition of the capacity to target proteins to different compartments has enabled eukaryotic cells to maintain and control the development of organelles. In higher plants the evolution of the TOC–TIC machinery has been a key mechanism enabling developmental processes. The evolutionary diversification of Toc receptors and transit peptides likely led to the tissue- and plastid type dependent preprotein selectivity of the import process. It is now well accepted that preprotein import in plastids plays a central role in the maintenance of cellular homeostasis, controlling the development and differentiation of this organelle. In a more indirect way, preprotein import also exerts control of nuclear gene expression via retrograde signaling to the nucleus. The composition and mode of action of the import machinery has been studied extensively in the past years, and now progress needs to be made toward the understanding of the regulatory mechanisms controlling the assembly and the activity of the complex. Regulation is not only important for correct sorting of preproteins, but also to limit energy expenditure associated with this costly process. Multiple types of PTM of Toc receptors have been discovered; however, their functional significance largely remains in the dark. Identification of the regulatory factors and signaling pathways as well as unraveling the biological relevance of the various PTM at the import machinery will provide new insight on how plants control development and adapt to the environment.

## Conflict of Interest Statement

The authors declare that the research was conducted in the absence of any commercial or financial relationships that could be construed as a potential conflict of interest.
